# *Dirofilaria immitis* in pinnipeds and a new host record

**DOI:** 10.1186/s13071-017-2073-0

**Published:** 2017-03-13

**Authors:** Ana Margarida Alho, Inês Marcelino, Vito Colella, Carla Flanagan, Nuno Silva, Jorge Jesus Correia, Maria Stefania Latrofa, Domenico Otranto, Luís Madeira de Carvalho

**Affiliations:** 1 CIISA, Faculdade de Medicina Veterinária, Universidade de Lisboa (ULisboa), Lisboa, Portugal; 2 Università degli Studi di Bari, Bari, Italy; 3 Mundo Aquático S.A. Zoomarine, Albufeira, Portugal

**Keywords:** *Dirofilaria immitis*, Pinnipeds, South African fur seal, Dirofilariosis, Vector-borne disease, Wildlife, Zoonosis

## Abstract

**Background:**

*Dirofilaria immitis* is a mosquito-borne pathogen that is spreading worldwide, and the associated infection (i.e. dirofilariosis) is becoming a threat to animals and humans living in endemic areas. Little is known about the occurrence and risk of infection of *D. immitis* in pinnipeds. Here we report dirofilariosis by *D. immitis* in several pinniped species kept in captivity in Portugal.

**Methods:**

Animals were housed in an oceanographic park located in Algarve, southern Portugal, a geographical area endemic for canine dirofilariosis. To assess the occurrence of *D. immitis*, blood was collected from the park’s resident pinniped population, which consisted of 16 animals (5 common seals *Phoca vitulina*, 2 grey seals *Halichoerus grypus*, 3 California sea lions *Zalophus californianus* and 6 South African fur seals *Arctocephalus pusillus pusillus*). *Dirofilaria immitis* nematodes were detected by real-time PCR and by the presence of circulating antigens. In addition, modified Knott’s technique was performed to detect circulating microfilariae. Necropsies and histopathological examination of two animals which died during the study were also conducted.

**Results:**

Out of the 16 pinnipeds housed at the park, seven (43.8%) were positive for *D. immitis* by real-time PCR (3 *P. vitulina*, 2 *Z. californianus* and 2 *A. p. pusillus*), two of which (*P. vitulina*) were also positive for the nematode’s antigen. Additionally, *D. immitis* microfilariae were detected in one *A. p. pusillus*. Furthermore, several *D. immitis* specimens were retrieved from the right ventricle and pulmonary arteries at the necropsy of one *P. vitulina* and one *A. p. pusillus*.

**Conclusions:**

This study provides new epidemiological data on *D. immitis* infection in pinnipeds diagnosed through clinical, molecular and pathological findings. Additionally, the South African fur seal is herein reported as a new host for this zoonotic filarioid. The situation herein described could also occur in other parks located in areas where canine dirofilariosis is endemic. Active surveillance and preventive measures of dirofilariosis in pinnipeds on a local and global scale are therefore vital to improve the early diagnosis and control of dirofilariosis.

## Background

*Dirofilaria immitis* (Spirurida: Onchocercidae) is a mosquito-borne pathogen spreading worldwide, and the associated infection (i.e. dirofilariosis) is becoming a threat to animals and humans living in endemic areas [1]. Although definitive hosts are primarily domestic and wild canids, *Dirofilaria immitis* shows low vertebrate host specificity, infecting several mammalian species (e.g. black bears, cats, ferrets, lions, otters, ocelots). In humans, this parasite may cause a severe clinical condition of increasing concern, with adult stages located mostly in the patient’s lungs, eyes or other anatomical districts [1]. However, little is known about the occurrence and risk of infection of *D. immitis* in pinnipeds. Only a few cases of infection in captive pinnipeds have been described so far [2–7]. Accordingly, no epidemiological studies on pinniped populations are available [8] and adult *D. immitis* have only been found in one hooded seal (*Cystophora cristata*) [2], one common seal (*Phoca vitulina*) [3], and in California sea lions (*Zalophus californianus*) kept in zoological parks in Florida [4], Louisiana [5] and Japan [6]. In the above-mentioned reports, nematodes were found upon necropsy in the right ventricle of the heart, pulmonary arteries, vena cavae, portal vein and pericardial sac [3–6]. Clinical signs, only documented in California sea lions, included cardiopulmonary impairment, coughing and laboured breathing [4, 5]. Indeed, pinnipeds might remain asymptomatic, even when large numbers of parasites inhabit their heart and associated vessels [3–6]. Here we report dirofilariosis in a population of pinnipeds housed at an oceanographic park in Portugal and the South African fur seal, *Arctocephalus pusillus pusillus*, as a new host for *D. immitis*.

## Methods

In 2013 and 2014, during the necropsy examinations of two adult South African fur seals (*A. p. pusillus*) housed at the Zoomarine park in Albufeira (Algarve, southern Portugal), *D. immitis* nematodes were accidentally found in the pulmonary arteries and right ventricle of two animals (animals A and B). These cases prompted an epidemiological survey to assess the occurrence of *D. immitis* in the park’s resident pinniped population (*n* = 16). In 2015, 5 common seals (*Phoca vitulina*), 2 grey seals (*Halichoerus grypus*), 3 California sea lions (*Zalophus californianus*) and 6 South African fur seals (*A. p. pusillus*) were surveyed for *D. immitis* infection. All animals were housed in facilities with pools and dry areas and no ecto- or endoparasitic treatments were administered. The animals originated from either Europe or Canada, and remained in Portugal for at least 10 years.

Physical examination was performed to check for the presence of abnormal clinical signs in individual animals. Blood was collected from the epidural intervertebral vein in the phocids (*P. vitulina* and *H. grypus*) and from the interdigital vein of the hind flippers or caudal gluteal vein in the otariids (*Z. californianus* and *A. p. pusillus*), as previously described [9]. A rapid commercial qualitative antigen WITNESS^®^ HW Heartworm Antigen Test Kit (Zoetis, Europe) was used to assess the presence of *D. immitis* circulating antigens and a modified Knott’s technique was performed to detect circulating microfilariae in the pinnipeds’ blood. In one of the animals, it was possible to perform an ultrasound to assess the presence of heartworm infection and evaluate cardiac function.

During this epidemiological survey two animals died and necropsies were conducted as described in [10, 11]. Lung and liver samples were collected for histopathological examination, fixed in 10% buffered formalin and embedded in paraffin; sections (3 μm thick) were stained with haematoxylin and eosin for routine microscopic examination.

Genomic DNA was extracted from segments (about 10 mm) of the adult worms collected from the necropsies and from the 16 blood samples, using a commercial kit (DNeasy Blood & Tissue Kit, Qiagen, GmbH, Hilden, Germany) and tested by real-time PCR (qPCR), based on SsoFast™ EvaGreen^®^, targeting partial cytochrome *c* oxidase subunit 1 (*cox*1), coupled with melting-curve analysis for the detection and discrimination of *Dirofilaria* spp. [12]. The real-time PCR products were purified using Ultrafree-DA columns (Millipore, Bedford, USA), sequenced using BigDye® Terminator v3.1 Cycle Sequencing Kit (Applied Biosystems Inc.) in an automated sequencer (ABI-PRISM 377; Applied Biosystems Inc.). All sequences generated were compared to sequences available in GenBank using Basic Local Alignment Search Tool (BLASTn) [13].

## Results

**Fig. 1:**
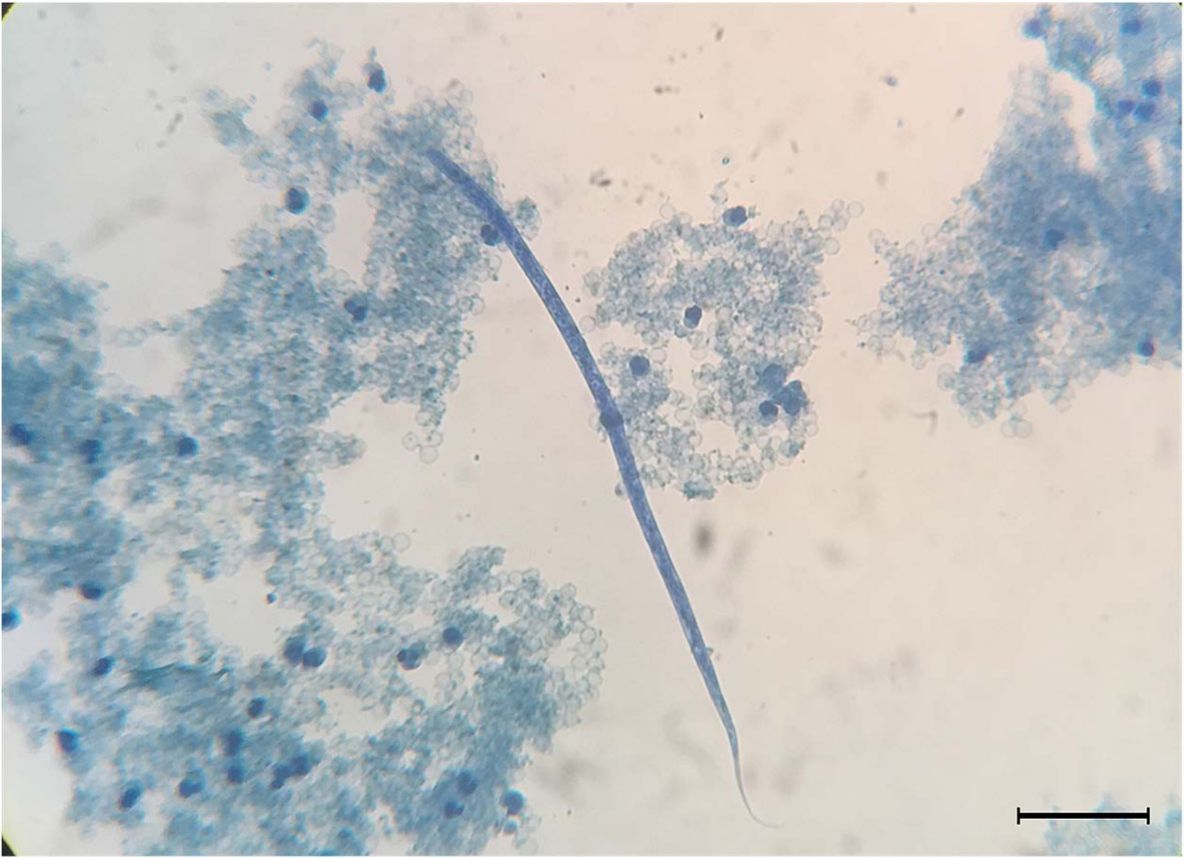
Microfilaria of *Dirofilaria immitis* detected using the modified Knott’s technique. *Scale-bar*: 50 μm

Two *P. vitulina* were antigen positive (12.5%) and one *A. p. pusillus* scored positive for *D. immitis* microfilariae (6.3%) (Table 1). Circulating microfilariae were 300–305 μm long bearing a conical anterior edge and straight rear end (Fig. 1). Seven (43.8%) out of the 16 animals were positive for *D. immitis* at qPCR (3 *P. vitulina*, 2 *Z. californianus* and 2 *A. p. pusillus*), with melting peaks (mean Tm = 75 °C) corresponding to the species-specific range of *D. immitis* positive control (mean ± SD: 75.7 ± 0.3 °C) (Table 1).

**Table 1:** Test results of the 16 pinnipeds surveyed to *Dirofilaria immitis*

Animal	Species	Sex	Country of origin	Birth year	Antigen test	Knott test	qPCR	Signs
1	*P. vitulina*	M	Portugal	2012	–	–	–	–
2	*P. vitulina*	F	Canada	1989	Positive	–	Positive^a^	–
3	*P. vitulina*	M	Canada	1996	–	–	–	–
4	*P. vitulina*	F	Portugal	2007	–	–	Positive	–
5	*P. vitulina*	F	Canada	1989	Positive	–	Positive	–
6	*H. grypus*	M	Portugal	2002	–	–	–	–
7	*H. grypus*	F	Canada	1990	–	–	–	–
8	*Z. californianus*	M	Portugal	1996	–	–	Positive	–
9	*Z. californianus*	M	Spain	2003	–	–	–	–
10	*Z. californianus*	M	Belgium	1996	–	–	Positive	–
11	*A. p. pusillus*	M	Portugal	1998	–	–	Positive	–
12	*A. p. pusillus*	M	UK	1992	–	Positive	Positive	Cough, lethargy and exercise intolerance
13	*A. p. pusillus*	M	Sweden	2002	–	–	–	–
14	*A. p. pusillus*	M	Sweden	2002	–	–	–	–
15	*A. p. pusillus*	M	Portugal	1996	–	–	–	–
16	*A. p. pusillus*	M	Spain	1996	–	–	–	–
Total		12M/4F			2	1	7	–

*Abbreviations*: *F* female, *M* male

^a^Animal in which transthoracic echocardiography was performed revealing the presence of linear mobile hyperechoic structures within the right ventricle and main pulmonary artery, consistent with heartworms

**Fig. 2:**
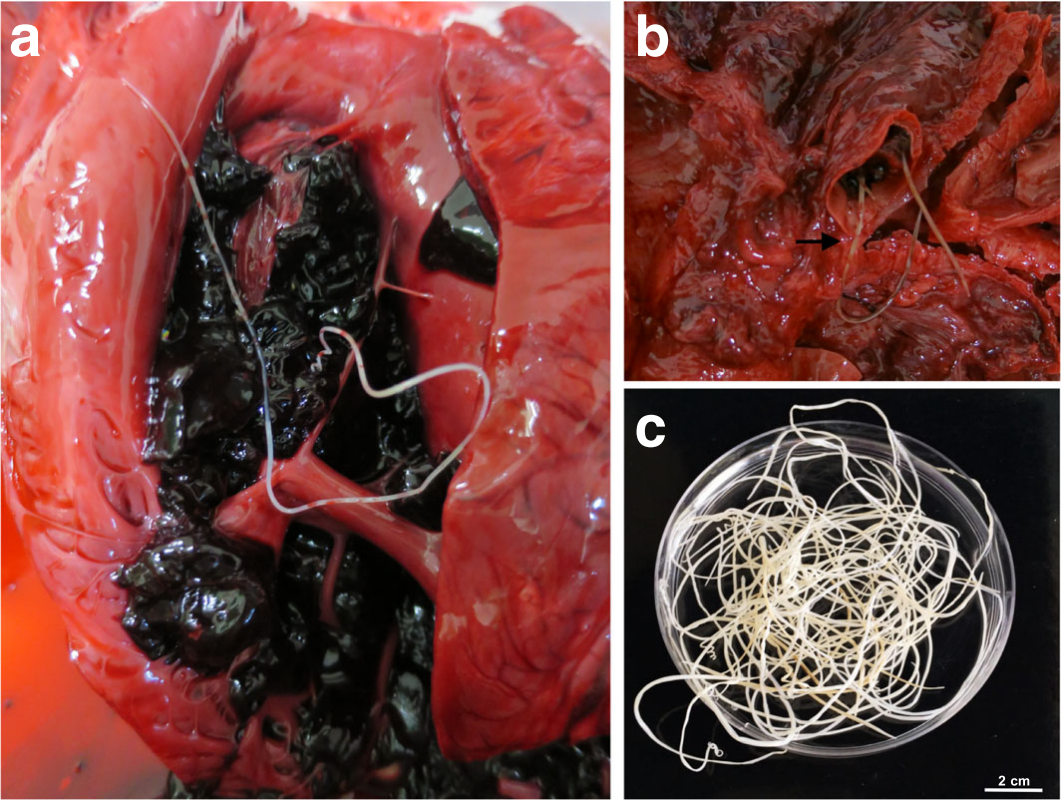
Adult nematodes of *Dirofilaria immitis* collected at the necropsies of South African fur seals. **a** Adult nematode in the right ventricle. **b** Adult nematodes (*arrow*) in the pulmonary artery, showing extensive pulmonary congestion. **c** Male and female adult nematodes recovered from the blood clot. *Scale-bar*: 2 cm

**Fig. 3:**
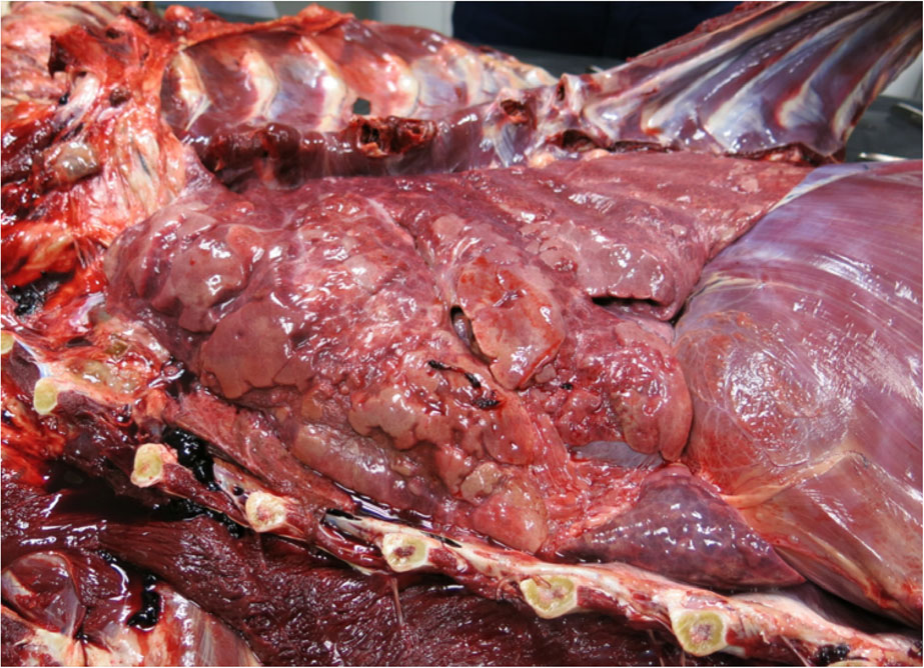
Macroscopic appearance of the lungs in the necropsy of a South African fur seal, highlighting an extensive pulmonary congestion and pulmonary haemorrhages

**Fig. 4:**
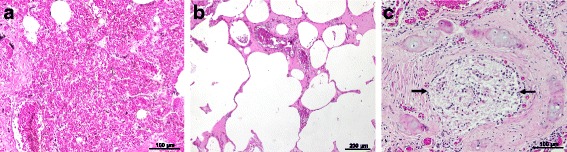
Histopathology of the lungs of a South African fur seal, stained with haematoxylin and eosin. **a** Severe pulmonary haemorrhages, note the presence of blood in the lumen of the alveoli. **b** Extensive pulmonary emphysema. **c** Catarrhal bronchitis, note the lumen of a bronchus obstructed with mucus (*arrows*)

One *P. vitulina* (Table 1, animal no. 5) and one *A. p. pusillus* (Table 1, animal no. 12) died during the study (Table 2). Data from these two animals were gathered with those obtained from animals’ A and B dead prior to the epidemiological survey. Overall, during the necropsies of these four animals (A, B, no. 5 and no. 12), adult male and gravid female nematodes were retrieved from the right ventricle and pulmonary arteries (Fig. 2). Macroscopically, lungs were congested and haemorrhagic, and mild right ventricular hypertrophy was noticed. In these four cases, gross and histopathological abnormalities associated with *D. immitis* infection were present, including pulmonary congestion and haemorrhages (Fig. 3), pulmonary emphysema, interstitial and exudative pneumonia, catarrhal bronchitis and hepatic congestion (Table 2, Fig. 4). In the four cases, cardiopulmonary impairment was noticed. The adult nematodes collected from the four individuals were morphologically and molecularly identified as *D. immitis*. All *cox*1 gene sequences obtained from the adult nematodes and from the pinniped’s blood were identical (GenBank accession number KX372755), showing 100% nucleotide identity to a *D. immitis* sequence in GenBank (KF553638).

**Table 2:** Data of the four necropsies of seals in which *Dirofilaria immitis* nematodes were detected

Animal ID	Species	Sex	Birth year	Death year	No. of adult *D. immitis*	Location of adult nematodes	Pathological observations and histological abnormalities
A	*A. p. pusillus*	M	1988	2013	15	Right ventricle and pulmonary artery	Severe pulmonary hemorrhages; extensive pulmonary congestion and moderate pulmonary emphysema
B	*A. p. pusillus*	M	1988	2014	10	Pulmonary artery	Moderate interstitial and exudative pneumonia; catarrhal bronchitis; moderate pulmonary congestion; central lobular hepatic congestion and dilation of the sinusoids
5	*P. vitulina*	F	1989	2016	32	Right ventricle and pulmonary artery	Extensive pulmonary congestion
12	*A. p. pusillus*	M	1992	2016	26	Right ventricle and pulmonary artery	Extensive pulmonary congestion

## Discussion

This study provides new epidemiological data of dirofilariosis by *D. immitis* in pinnipeds, diagnosed through clinical, molecular and pathological findings. The South African fur seal is herein described as a new host of *D. immitis*. All animals were housed in a tourist attraction (i.e. an oceanographic park) in the Algarve region, southern Portugal, an area that records the highest number of days/year with suitable conditions for *Dirofilaria* transmission [14], and the highest prevalence of canine dirofilariosis in mainland Portugal [15].

For the first time, qPCR was successfully used to diagnose *D. immitis* infection in pinnipeds. Indeed, qPCR detected seven infected animals, of which two *P. vitulina* and one *A. p. pusillus* which were also positive for circulating antigen and microfilariae, respectively. qPCR was highly sensitive in diagnosing *D. immitis* in pinnipeds, since it was able to detect one animal that was only antigen-positive (no. 5) and another that was only-microfilaremic (no. 12), although both presented several nematodes (including gravid females) during the necropsy. In addition, it detected also another case (animal no. 2) only positive for the antigen, but in which transthoracic echocardiography revealed linear hyperechoic structures consistent with heartworms within the right ventricle and main pulmonary arteries. Furthermore, qPCR was also able to detect four other animals that were negative in all other diagnostic tests used (microfilariae and/or antigen; Table 1). All pinnipeds were retested by modified Knott’s technique and antigen test to rule out potential false negatives. These further analyses displayed identical results. Positivity to qPCR suggests that alive parasites have been in contact with pinnipeds with no information on the current infection status. Indeed, this molecular assay may detect *D. immitis* DNA from a current infection or, theoretically, from a recently cleared infection. In addition, dirofilariosis in pinnipeds may be featured by transient and low intensity microfilaremia, as in the case of infection in cats. This might be the reason for the detection of microfilaremia in only one of the two pinnipeds who had male and female nematodes at necropsies. Additionally, rapid commercial *D. immitis* antigen tests were specifically developed for canine and feline blood samples, thus, their sensitivity and performance might be poor when used in pinnipeds, underestimating the true prevalence.

Although pinnipeds are aquatic mammals, they spend large periods of their life in terrestrial environments, and are therefore exposed to mosquito bites. As in the present survey, *D. immitis* could also occur in other parks in countries with endemic areas for canine dirofilariosis. The occurrence of this situation in the Algarve region, a popular summer destination, should be carefully considered due to the zoonotic potential of this parasite. Indeed, although human dirofilariasis has been often underdiagnosed (probably due to the lack of awareness amongst health professionals and to the difficulties in parasite identification), two cases of pulmonary nodules by *D. immitis* [16] and two cases of subcutaneous dirofilariasis by *Dirofilaria repens* [17, 18] have already been reported in Portugal.

## Conclusions

This study emphasizes the need for active surveillance of dirofilariosis in facilities where animals and humans are in close contact, and strengthens the need for routine heartworm preventive measures [19] and vector control strategies. The high prevalence of *D. immitis* herein reported in a confined area where pinnipeds are kept, may represent a risk interface for zoonotic pathogen transmission. Therefore, a One Health approach applied on a local and global scale [20] is vital to improve early diagnosis and control of zoonotic pathogens in humans and wildlife.

## Abbreviation

qPCR: real-time PCR
